# Impact of Age and BMI on Spinal Anesthesia Characteristics in Pediatric Patients: A Retrospective Study

**DOI:** 10.3390/medicina61101792

**Published:** 2025-10-03

**Authors:** Ahmet Atlas, Nuray Altay, Evren Büyükfirat, Abdulhakim Şengel, Ramazan Aslanparçası, Abdullah Şengül

**Affiliations:** 1 Department of Anesthesiology and Reanimation, Faculty of Medicine, Harran University, 63040 Şanlıurfa, Turkey; ahmetatlas@harran.edu.tr (A.A.); nurayaltay@ymail.com (N.A.); evrenbf@gmail.com (E.B.); 2 Şanlıurfa Mehmet Akif İnan Eğitim ve Araştırma Hastanesi, 63040 Haliliye, Turkey; r_hacettepe@hotmail.com (R.A.); abdullahsengul342@gmail.com (A.Ş.)

**Keywords:** pediatric anesthesia, spinal block, sensory block duration, BMI, sedation, bupivacaine, regional anesthesia

## Abstract

*Background and Objectives*: Spinal anesthesia is increasingly preferred in pediatric surgeries due to its rapid onset, high success rate, and low risk of systemic complications. However, the influence of age and body mass index (BMI) on block characteristics in adolescents remains insufficiently studied. *Materials and Methods*: This retrospective study evaluated 190 pediatric patients (aged 9–18 years; 154 male, 36 female) undergoing elective surgery with spinal anesthesia. Patients were stratified by age (Group 1: 9–14 years; Group 2: 15–18 years) and BMI (Group A: 16.00–19.65 kg/m^2^; Group B: 19.66–23.31 kg/m^2^). The primary outcome was sensory block duration. Secondary outcomes included sedation requirements, complications, analgesic requirement times, and Visual Analog Scale (VAS) scores. *Results*: Group 2 had significantly longer sensory block duration and lower postoperative VAS scores at 3 and 12 h compared to Group 1 (*p* < 0.001). Lower BMI was associated with greater sedation requirements (*p* < 0.001) and a higher incidence of intraoperative nausea and vomiting (*p* = 0.013). Complications were infrequent (hypotension 6.3%, bradycardia 2.1%, PONV 7.1%, postoperative headache 3.1%) and managed conservatively. *Conclusions*: Age and BMI meaningfully influence spinal anesthesia characteristics in pediatric patients. Older adolescents achieved longer sensory block durations and better postoperative analgesia, whereas younger and lower-BMI patients required more sedation and had higher nausea rates. Individualized spinal anesthesia planning, considering age, BMI, and developmental stage, may optimize clinical outcomes. Prospective studies are warranted to validate these findings.

## 1. Introduction

Spinal anesthesia has long been utilized as an effective and safe alternative to general anesthesia in pediatric surgeries. Its rapid onset of action, high success rate, and minimal systemic side effects make it a preferred technique, particularly for short-duration procedures involving the lower extremities and lower abdominal regions [1,2]. In pediatric patients, the limited physiological reserve and the increased risk of complications associated with airway manipulation further highlight the clinical reliability of spinal anesthesia [3].

In recent years, growing concerns about the potential long-term neurodevelopmental effects of general anesthesia administered during childhood have led to increased interest in regional anesthesia techniques. The U.S. Food and Drug Administration (FDA) has cautioned that prolonged or repeated exposure to general anesthesia—especially for durations exceeding three hours—may adversely affect the developing brain [4]. Consequently, minimally invasive techniques such as spinal anesthesia have gained priority, particularly in elective and short-duration surgical interventions [5]

The safe use of spinal anesthesia in children has been reported across a wide age range, from infancy to adolescence [6,7]. However, the current literature predominantly focuses on neonates and young children, offering limited data on the adolescent population [8]. Children aged 9 to 18 represent a heterogeneous group in anatomical and physiological development. In this study, the 9–14 age range was defined as early adolescence and the 15–18 age range as late adolescence, following developmental medicine classifications and prior anesthetic literature that report meaningful differences in cooperation, anatomical landmarks, and cerebrospinal fluid dynamics between these age groups [9,10]. Dividing the cohort into these two groups may allow for a more nuanced evaluation of spinal block characteristics and sedation needs.

In addition, patients were further classified into two BMI categories: 16.00–19.65 kg/m^2^ and 19.66–23.31 kg/m^2^. These thresholds were selected to represent lower- and higher-normal BMI ranges for the adolescent age group, based on WHO growth reference data and previous anesthetic studies assessing block spread and sedation response in relation to body composition [11,12]. This classification aimed to explore whether subtle differences within the normal BMI spectrum influence spinal anesthesia performance and clinical outcomes. Furthermore, adolescents (9–18 years) constitute a heterogeneous group in terms of anatomy, CSF volume, and cooperation, which can influence spinal block characteristics and sedation needs.

In this context, we retrospectively evaluated 190 pediatric patients who underwent spinal anesthesia. This study aimed to investigate the effects of variables such as age and BMI on the process and duration of spinal anesthesia and identify any potential differences. The primary hypothesis was that age group (9–14 vs. 15–18 years) would significantly influence spinal block duration and sedation requirements. The secondary hypothesis was that BMI would affect the volume of local anesthetic administered, sensory block duration, and the need for sedation. This study aims to address a gap in the current literature and to provide data that may inform and guide clinical practice. The secondary hypothesis was that BMI, even within a normal range, may influence clinical and anesthetic outcomes, specifically the volume of local anesthetic administered, the duration of sensory block, and the likelihood of requiring sedation.

## 2. Materials and Methods

### 2.1. Study Design

This retrospective, single-center, observational study was conducted after obtaining approval from the Harran University Clinical Research Ethics Committee (Date: 11 December 2023, Approval No: 23/23/18). Medical records of pediatric patients aged 9–18 years who underwent spinal anesthesia between October 2020 and April 2023 were reviewed. Given the retrospective nature, there is a potential for selection bias and unmeasured confounding. However, these limitations were mitigated by applying predefined inclusion/exclusion criteria and stratifying the analysis according to both age and BMI groups.

Data abstraction followed a standardized case report form, and two investigators independently verified entries against source charts to minimize transcription errors and selection bias.

### 2.2. Participants

A total of 190 pediatric patients with American Society of Anesthesiologists (ASA) physical status classification I or II who underwent elective surgical procedures under spinal anesthesia were included. ASA I was defined as healthy patients without systemic disease, and ASA II as patients with mild systemic disease without functional limitation. Surgical procedures included orthopedic, urological, and lower abdominal surgeries. Subgroup analysis revealed no significant differences across surgery types regarding block duration or sedation requirements (all *p* > 0.05).

Exclusion criteria: ASA III–V status, severe inflammatory or immunological disorders, cognitive or neurological dysfunction, allergy to local anesthetics, history of paresthesia or motor nerve injury.

Age groups: Patients were stratified into two age groups based on developmental stages. Group 1 included patients aged 9–14 years, representing early adolescence, whereas Group 2 included patients aged 15–18 years, representing late adolescence. This classification was adopted to capture potential differences in spinal block characteristics related to anatomical and physiological changes across adolescence [9,10].

BMI groups: BMI was categorized into two ranges according to WHO growth reference data and previous anesthetic literature: Group A (16.00–19.65 kg/m^2^, lower-normal range) and Group B (19.66–23.31 kg/m^2^, higher-normal range) [11,12]. This stratification allowed for exploration of whether subtle variations within the normal BMI spectrum could influence spinal anesthesia performance and clinical outcomes.

### 2.3. Anesthesia Protocol

After admission to the preoperative unit, standard monitoring (non-invasive blood pressure, SpO_2_, heart rate, and intravenous fluid infusion) was established. Spinal anesthesia was performed under aseptic conditions, with patients positioned in either the sitting or lateral decubitus position depending on age and cooperation.

All procedures were performed using a midline approach under aseptic conditions. The puncture level was L4–L5 for patients younger than 14 years and L3–L4 for those aged 15 years and older, consistent with anatomical safety recommendations.

All spinal blocks were performed by experienced anesthesiologists from the same department; however, individual operator variability could not be fully eliminated.

The local anesthetic was 0.5% hyperbaric bupivacaine (Heavy Marcaine), with dose determined by height (<150 cm → 1.5 mL; 150–165 cm → 2.0 mL; 165–180 cm → 2.5 mL; >180 cm → 3.0 mL). A height-based approach was preferred over BMI-based dosing because height correlates more directly with spinal canal length and cerebrospinal fluid volume, ensuring more consistent intrathecal spread [13,14]. This strategy allowed analysis of BMI effects independent of dosing differences, although it may have simultaneously attenuated variability attributable to BMI, potentially reducing the ability to detect BMI-related influences on block spread and duration.

### 2.4. Sedation Protocol

Sedation administration followed institutional routine practice without a standardized protocol, potentially introducing variability in clinical decisions. Baseline anxiety was not assessed with a validated scale (e.g., mYPAS), which limited objective comparison of sedation requirements.

When sedation was required, intravenous midazolam (0.05 mg/kg) was administered. The decision was at the discretion of the attending anesthesiologist, based on patient anxiety, cooperation, and surgical conditions, and the dose and timing were recorded from anesthesia charts.

### 2.5. Outcomes

The primary outcome was sensory block duration (minutes) from intrathecal injection until regression of sensory block by two dermatomes.

Secondary outcomes included preoperative and intraoperative sedation requirement (yes/no); motor block duration; volume of local anesthetic used; time to first analgesic requirement (hours); Visual Analog Scale (VAS) scores at 1, 3, 6, and 12 postoperative hours; intraoperative and postoperative complications (hypotension, bradycardia, nausea, vomiting, headache, urinary retention, neurological deficit); and surgeon and parental satisfaction scores.

### 2.6. Statistical Analysis

Effect sizes and 95% confidence intervals are reported for key comparisons, and secondary outcomes are consistently interpreted as exploratory. The post hoc power analysis for the primary outcome was based on the observed mean difference in sensory block duration between age groups (Cohen’s d = 0.48, 95% CI: 0.19–0.77), indicating a moderate effect size.

Normality was tested using the Kolmogorov–Smirnov test. Non-normally distributed continuous variables were analyzed with the Mann–Whitney U test, and categorical variables with chi-square or Fisher’s exact tests, as appropriate. Considering the number of variables analyzed, results for secondary outcomes should be interpreted as exploratory. A *p*-value of <0.05 was considered statistically significant. Statistical analyses were conducted using IBM SPSS Statistics version 26.0 and Microsoft Excel (Office 16).

## 3. Results

A total of 190 patients were included in the study. Patients were divided into two groups based on age and BMI. Table 1 presents the demographic characteristics of pediatric/adolescent patients who underwent spinal anesthesia. This gender imbalance reflects the underlying distribution of surgical indications at our institution—particularly circumcision and inguinal hernia repair—which predominantly involve male patients. Subgroup analysis showed no significant gender effect on sensory block duration or sedation requirements.

A statistically significant difference was found between the age groups (Group 1: 9–14 years, Group 2: 15–18 years) regarding pre-procedural sedation requirement, patient positioning during the procedure, number of needle redirections, and volume of local anesthetic administered (all *p* < 0.05). No significant difference was observed between the groups regarding cold sensation test results or the type of surgical procedure performed (*p* ≥ 0.05). Pre-procedural sedation requirement and the frequency of multiple needle redirections were significantly higher in Group 1 compared to Group 2 (both *p* < 0.001). Furthermore, the lateral decubitus position was used more often in Group 1, whereas the sitting position predominated in Group 2 (*p* < 0.001). The volume of local anesthetic administered was significantly higher in Group 2 (*p* < 0.001) (Table 2).

A statistically significant difference was also found between the groups regarding intraoperative additional sedation requirements (*p* < 0.001), with Group 1 requiring more frequent sedation. No significant differences were observed in intraoperative hypotension, bradycardia, nausea and vomiting, conversion to general anesthesia, duration of surgery, or surgeon satisfaction (*p* ≥ 0.05) (Table 2).

To contextualize these findings, between age groups, the absolute difference in mean sensory block duration was Δ = 173.2 − 165.6 = 7.6 min, and time to first analgesic differed by Δ = 0.16 h (≈9.5 min), highlighting the clinical impact beyond statistical significance. Sensory block duration and time to first analgesic requirement were both significantly longer in Group 2 (*p* < 0.001 and *p* = 0.014, respectively). Motor block duration did not differ significantly between groups (*p* = 0.665). VAS scores at 3 and 12 h postoperatively were significantly lower in Group 2, indicating better analgesic control (*p* = 0.002 and *p* < 0.001, respectively). VAS1 scores were identical (fixed at 1.00) for all patients, as the initial postoperative pain assessment was performed in the recovery room while the spinal block was still dense. This uniform score reflects the timing of measurement rather than data entry error. No significant difference was observed in VAS6 scores (*p* = 0.822) (Table 2).

Postoperative neurological complications were absent in all patients at one-week follow-up, and attendance rates at this visit did not differ between groups. Adverse events after the first postoperative week were rare and did not differ significantly (Table 2).

**Figure 1:**
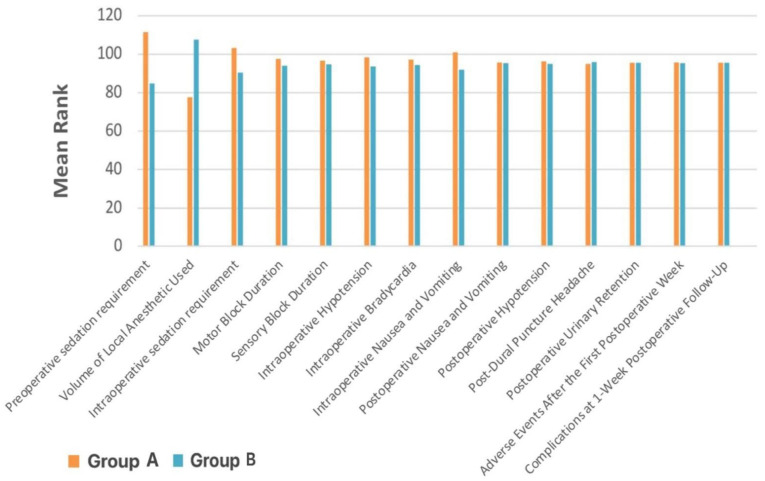
Variation in the analyzed parameters between BMI Group A (16.00–19.65 kg/m^2^) and BMI Group B (19.66–23.31 kg/m^2^). Data are presented as mean rank.

When stratified by BMI, there were no significant differences between Group A (16.00–19.65 kg/m^2^) and Group B (19.66–23.31 kg/m^2^) in terms of sensory or motor block duration, intraoperative sedation, or most postoperative complications (*p* ≥ 0.05) (Table 3, Figure 1). However, Group A required significantly more preoperative sedation (*p* < 0.001), had a higher incidence of intraoperative nausea and vomiting (*p* = 0.013), and received a smaller volume of local anesthetic (*p* < 0.001).

## 4. Discussion

This retrospective study aimed to evaluate factors influencing spinal anesthesia’s duration and postoperative effects in pediatric patients aged 9 to 18 years. Our findings demonstrated that variables such as age and BMI significantly affect spinal block characteristics. In particular, the 15–18-year age group exhibited a significantly longer duration of sensory block than the 9–14-year group (*p* < 0.001). This finding highlights the need for spinal anesthesia to be tailored to individual characteristics in pediatric and adolescent populations, emphasizing the importance of careful consideration of technique in this age group.

The choice of technique and positioning for spinal anesthesia in children can be evaluated similarly to adults. The literature reports that lumbar puncture in pediatric patients can be performed in either the sitting or lateral decubitus position [9,10]. In our study, lumbar puncture was performed in the lateral decubitus position in the 9–14-year group and in the sitting position in the 15–18-year group. This preference likely reflects the difficulty communicating with younger children and the greater ease of positional cooperation in older adolescents.

The entry level for spinal anesthesia may vary with age due to anatomical development. Previous studies have indicated that the L4–L5 interspace is safer in children, while the L3–L4 level is more appropriate in adults. This difference arises from the conus medullaris typically terminating at the L3 level in children, which may increase the risk of spinal cord injury when accessing the L3–L4 level [1,11]. As age increases, the conus ascends to the L1–L2 level, making the L3–L4 interspace safer for needle insertion. These anatomical considerations underscore the importance of age-appropriate planning of the entry level. In a study by Kokki et al. (1998), it was suggested that the spinal entry level may influence both the success of the procedure and block duration [12]. Accordingly, our study used the L4–L5 interspace for patients under 14 years of age and the L3–L4 level for those aged 15 years and older.

In pediatric spinal anesthesia, local anesthetic doses are typically calculated based on body weight (mg/kg). However, parameters such as cerebrospinal fluid (CSF) volume, spinal column length, and intrathecal space capacity are influenced by both weight and height. Several studies have suggested that weight-based dose calculations in younger children may fail to achieve sufficient block levels due to variability in the intrathecal space [13]. Other studies have shown that height is a more reliable determinant for local anesthetic dosing, as it correlates better with intrathecal volume [14]. Based on this rationale, our study applied a height-based dosing regimen: patients <150 cm received 1.5 mL, those 150–165 cm received 2.0 mL, those 165–180 cm received 2.5 mL, and those >180 cm received 3.0 mL of local anesthetic.

Age-related differences in block duration may reflect developmental changes in lumbosacral CSF volume, spinal canal length, and intrathecal distribution dynamics, which can alter the pharmacodynamic properties of local anesthetics during adolescence. Consistent with these mechanisms, our findings showed that patients in the 15–18-year group had significantly longer sensory block durations than those in the 9–14-year group. Although this observation contradicts some earlier literature, it supports the notion that spinal block duration does not follow a simple linear decrease with increasing age. During adolescence, changes in CSF composition, spinal canal depth, and intrathecal distribution patterns may modulate block characteristics. In a study by Hannan et al. on children undergoing laparoscopic appendectomy under spinal anesthesia, longer motor block durations were observed in older children [15]. Similarly, while the study by Çalışkan et al. did not directly compare block durations across age groups, it was clinically noted that spinal blocks were more stable and longer-lasting in patients older than 10 years [8]. Together, these findings suggest that anatomical development and technical factors interact to shape the relationship between age and block duration.

Significant differences were also observed between the 9–14 and 15–18 age groups regarding spinal anesthesia’s technical feasibility and clinical effectiveness. The younger age group required more pre-procedural sedation, more needle redirections, and demonstrated greater procedural difficulty. These challenges may be attributed to smaller and more variable anatomical structures, higher baseline anxiety levels, and limited cooperation due to immature cognitive and emotional development.

Sedation requirements prior to spinal anesthesia were significantly associated with both age and BMI (*p* < 0.05 for all). Specifically, younger and lower-BMI patients demonstrated a higher need for sedation. This may be explained by developmental vulnerability to anxiety, heightened sensitivity to environmental stressors, and limited communication skills. Inadequate psychological preparation, fear of the procedure, and hyperactive behaviors commonly observed in younger children are additional contributors [16]. In a 2019 survey by Rehfuss et al., pediatric surgeons and anesthesiologists emphasized sedation as an important supportive tool for both patient comfort and surgical team satisfaction [4]. However, deep sedation may diminish paresthesia awareness and increase the risk of overlooking potential neurological complications [13]. Therefore, sedation in younger and lower-BMI patients should be carefully titrated, administered at an appropriate depth, and monitored continuously.

In contrast, the volume of local anesthetic administered was significantly higher in the 15–18 age group, which corresponded with a longer sensory block duration. This supports the view that increased CSF volume and a wider spinal canal in older patients necessitate larger volumes to achieve effective blockade. Additionally, the time to first analgesic requirement was significantly longer in this age group, indicating that spinal anesthesia provided more prolonged analgesic efficacy and superior postoperative pain control.

Evaluation of VAS (Visual Analog Scale) scores revealed significantly lower pain scores at both the 3rd and 12th postoperative hours in the older age group. This result suggests that spinal block lasted longer and provided more effective analgesia in older children. The literature also reports a positive correlation between increasing age and both the duration and effectiveness of spinal anesthesia [8,15]. In our study, the significantly lower VAS scores at the 3rd hour in the older group further support the delayed onset of analgesic need in these patients.

In conclusion, age is a critical factor influencing both spinal anesthesia’s technical feasibility and analgesic efficacy. While younger children present more technical challenges and require more sedation, older children experience longer-lasting and more effective spinal blocks. Therefore, spinal anesthesia should be individualized according to age-specific considerations. In particular, technical preparation, psychological support, and sedation strategies must be carefully planned for younger patients to ensure safe and effective anesthesia.

Cardiorespiratory changes associated with spinal anesthesia occur less frequently in pediatric patients than in adults [17]. In particular, studies focusing on infants have indicated that due to reduced sympathetic nervous system dominance, the hemodynamic response to spinal anesthesia is often minimal [16,18]. However, in children aged five years and older, bradycardia and hypotension may occur following spinal anesthesia [9]. In a study by Çalışkan et al., the incidence of bradycardia was reported as 1.1%, and hypotension as 2.3%, with the latter often associated with deep sedation [8].

In the present study, bradycardia was observed in 4 patients and hypotension in 11 patients. One of the known complications of spinal anesthesia, post-dural puncture headache, is observed less frequently in pediatric patients compared to adults [19]. In adults, the reported incidence ranges from 0.4% to 5% [20,21]. In a study by Imbelloni et al., including 307 children under 13 years of age, the incidence of post-dural puncture headache was 0.9% when using a 26-gauge spinal needle [10]. In our study, postoperative headache was reported by 6 patients, all of whom responded successfully to conservative treatment.

Regarding postoperative nausea and vomiting (PONV), Verma et al. reported no vomiting episodes among 102 pediatric patients aged 6 months to 14 years who underwent various surgical procedures under spinal anesthesia [2]. Similarly, in a study by Ahmed et al. involving 78 children, 6 cases of nausea and 1 case of vomiting were documented [22]. In our cohort, only 14 out of 190 patients experienced intraoperative nausea, and no cases of vomiting were recorded. The literature indicates that such adverse effects in pediatric patients are typically manageable with conservative treatment strategies [23,24,25]. Likewise, the low-incidence side effects observed in our study were successfully managed using non-invasive methods.

These findings support the safety profile of spinal anesthesia in pediatric patients concerning complication rates. Adverse events such as nausea, vomiting, bradycardia, hypotension, and headache were infrequent and successfully managed without invasive interventions. Our study aligns with the existing literature reporting low complication rates for spinal anesthesia in children [8]. Importantly, no patient experienced multiple severe complications.

Furthermore, when patients were stratified according to BMI, significant differences were observed regarding pre-procedural sedation requirements, volume of local anesthetic administered, and incidence of intraoperative nausea and vomiting. These findings highlight the impact of body composition on the spinal anesthesia process and suggest that BMI is an important factor to consider in pediatric anesthesia management.

The significantly higher sedation requirements among children with lower BMI values may be attributed to their generally younger age, higher anxiety levels, and limited procedural cooperation. Research also supports the increased need for sedation in children with low BMI, associating it with younger age, behavioral immaturity, and reduced coping capacity with medical interventions [26].

Similarly, the significantly higher incidence of intraoperative nausea and vomiting in the low BMI group may be linked to increased susceptibility to vagal stimulation and hypotension following sympathetic blockade. Additionally, these children often have reduced intravascular volume reserves, making them less adaptable to abrupt hemodynamic shifts induced by spinal blockade. Previous studies have suggested that nausea and vomiting associated with spinal anesthesia are correlated with physiological factors such as low BMI and hypovolemia, and emphasize the need for safe sedation strategies and prophylactic antiemetic measures in this population [27].

On the other hand, patients with higher BMI values were found to have received significantly greater volumes of local anesthetic. This finding may be explained by expanding the intrathecal space in parallel with increasing body weight, which necessitates higher anesthetic volumes to achieve effective blockade [28].

These findings suggest that BMI should be taken into account when planning spinal anesthesia. In particular, for patients with low BMI, the need for sedation support and prophylaxis against nausea and vomiting should be prioritized to minimize the risk of complications. In our study, BMI did not significantly affect block duration. However, in adult patients, it is known that BMI can influence the level and spread of spinal block through its relationship with cerebrospinal fluid (CSF) volume [29]. The absence of a clear correlation in pediatric patients may be due to the relatively lower impact of BMI on CSF volume in this population. Further research is needed to better elucidate this relationship in children.

In a case series conducted by Hannan et al. investigating pediatric laparoscopic appendectomy cases, patients who received spinal anesthesia were shown to experience greater postoperative comfort, shorter hospital stays, and reduced treatment costs [30]. Similarly, spinal anesthesia stood out in our study with its high success rate, stable hemodynamic profile, and low complication incidence.

## 5. Limitations

This study has several limitations. First, due to its retrospective design, patient data were collected from existing medical records, which limited the ability to standardize and comprehensively monitor certain clinical variables. This design also carries an inherent risk of selection bias and unmeasured confounding, as patient allocation to age and BMI groups was based on existing demographics rather than randomization.

Second, the study did not include a formal preoperative anxiety or sedation scale, such as the Modified Yale Preoperative Anxiety Scale (mYPAS), which could have provided an objective assessment of psychological factors influencing sedation requirements. Sedation administration was based on clinician judgment, introducing potential inter-operator variability. Similarly, while all spinal blocks were performed by experienced anesthesiologists, individual technical differences may have influenced block characteristics.

Third, the relatively narrow BMI range (16.00–23.31 kg/m^2^) and the predominance of normal-weight patients may have reduced the ability to detect a broader spectrum of BMI-related effects on spinal block characteristics. This limitation likely contributed to the absence of a significant correlation between BMI and block duration.

Fourth, spinal block level and spread were not verified using imaging methods such as ultrasound or fluoroscopy, which limits the anatomical precision of block assessment. Block level was determined clinically, which is standard in practice but may be less precise in research settings.

Fifth, although the local anesthetic type was standardized, dosing was height-based, which intentionally minimized BMI influence but may limit comparability with studies using weight-based dosing strategies.

Finally, the single-center nature of this study and the moderate sample size may limit generalizability. Larger, prospective, multicenter studies with standardized sedation assessment, broader BMI distribution, and imaging confirmation of block level are needed to validate and expand upon these findings.

## 6. Conclusions

Based on the findings of this single-center retrospective study, this study supports the safety of spinal anesthesia in pediatric patients aged 9–18 years, with age and BMI exerting measurable effects on block characteristics and sedation requirements. Older adolescents demonstrated longer sensory block durations, lower postoperative pain scores, and delayed need for rescue analgesia, while younger and lower-BMI patients required more frequent preoperative sedation and experienced a slightly higher incidence of intraoperative nausea.

These observations provide clinical suggestions for individualized spinal anesthesia planning, taking into account patient age, BMI, and developmental stage to optimize both intraoperative management and postoperative recovery. However, given the retrospective design, narrow BMI range, and absence of objective sedation scoring, these results should be interpreted with caution.

Future prospective multicenter studies incorporating standardized anxiety and sedation assessments, a wider range of BMI values, and imaging confirmation of block level are needed to confirm these associations and refine dosing and sedation strategies for pediatric patients.

## Figures and Tables

**Table 1:** Demographic characteristics of the patients according to age groups.

Variable	Group		n	mean	min	max	*p*
Age	Group 1		95	11.747	9	14	0.00
	Group 2		95	16.515	15	18	
Height	Group 1		95	154.252	125	172	0.00
	Group 2		95	172.894	162	184	
Weight	Group 1		95	46.957	29	58	0.00
	Group 2		95	60.842	50	76	
Gender	Group 1	Male	77				1.00
		Female	18				
	Group 2	Male	77				
		Female	18				
ASA	1	Group 1	87				0.47
		Group 2	84				
	2	Group 1	8				
		Group 2	11				

Data are presented as mean, minimum (min), and maximum (max) values. Group 1 = 9–14 years; Group 2 = 15–18 years. *p*-values are based on the Kolmogorov–Smirnov normality test. Abbreviations: ASA = American Society of Anesthesiologists; VAS = Visual Analog Scale; BMI = body mass index.

**Table 2:** Comparison of the evaluated parameters between age groups.

Parameter	Group	n	Mean Rank	95% CI	*p*
Preoperative Variables					
Preoperative sedation requirement	Group 1	95	117.50	0.77–0.91	0.000 *
	Group 2	95	73.50	0.30–0.49	
Patient position during procedure	Group 1	95	114.00	0.35–0.54	0.000 *
	Group 2	95	77.00	0.02–0.12	
Number of needle redirections	Group 1	95	102.44	1.50–1.85	0.044 *
	Group 2	95	88.56	1.29–1.57	
Volume of local anesthetic used	Group 1	95	55.74	2.36–2.49	0.000 *
	Group 2	95	135.26	3.02–3.12	
Cold sensation test	Group 1	95	92.50	0.06–0.18	0.097
	Group 2	95	98.50	0.02–0.10	
Type of surgical procedure	Group 1	95	90.01	nan–nan	0.069
	Group 2	95	99.97	nan–nan	
**Intraoperative Variables**					
Intraoperative sedation requirement	Group 1	95	112.00	0.48–0.67	0.000 *
	Group 2	95	79.00	0.16–0.33	
Intraoperative hypotension	Group 1	95	93.50	0.02–0.10	0.234
	Group 2	95	97.50	0.04–0.16	
Intraoperative bradycardia	Group 1	95	95.50	0.01–0.07	1.000
	Group 2	95	95.50	0.01–0.07	
Intraoperative nausea and vomiting	Group 1	95	95.50	0.04–0.14	1.000
	Group 2	95	95.50	0.04–0.14	
Need for general anesthesia	Group 1	95	95.50	0.00–0.00	1.000
	Group 2	95	95.50	0.00–0.00	
Duration of surgery	Group 1	95	90.15	37.15–44.49	0.175
	Group 2	95	100.85	43.00–53.40	
Surgeon satisfaction	Group 1	95	96.48	3.71–3.89	0.718
	Group 2	95	94.52	3.68–3.87	
Motor block duration	Group 1	95	93.79	130.18–135.50	0.665
	Group 2	95	97.21	132.36–135.74	
Sensory block duration	Group 1	95	81.49	162.00–169.16	0.000 *
	Group 2	95	109.51	170.35–176.07	
**Postoperative Outcomes**					
Postoperative nausea and vomiting	Group 1	95	95.00	0.01–0.09	0.701
	Group 2	95	96.00	0.02–0.10	
Postoperative hypotension	Group 1	95	94.50	0.01–0.07	0.408
	Group 2	95	96.50	0.02–0.10	
Post-dural puncture headache	Group 1	95	94.50	0.01–0.07	0.408
	Group 2	95	96.50	0.02–0.10	
Postoperative urinary retention	Group 1	95	95.50	0.00–0.00	1.000
	Group 2	95	95.50	0.00–0.00	
Time to first analgesic requirement	Group 1	95	88.00	3.11–3.27	0.014 *
	Group 2	95	103.00	3.25–3.44	
Parental satisfaction	Group 1	95	90.01	4.16–4.41	0.125
	Group 2	95	100.99	4.28–4.54	
VAS1	Group 1	95	95.50	1.00–1.00	1.000
	Group 2	95	95.50	1.00–1.00	
VAS3	Group 1	95	107.08	3.85–4.42	0.002 *
	Group 2	95	83.92	3.25–3.83	
VAS6	Group 1	95	96.27	2.45–2.66	0.822
	Group 2	95	94.73	2.45–2.65	
VAS12	Group 1	95	112.70	2.92–3.16	0.000 *
	Group 2	95	78.30	2.43–2.77	
Postoperative 1-week follow-up attendance	Group 1	95	96.50	0.08–0.22	0.663
	Group 2	95	94.50	0.07–0.20	
Adverse events observed after the first postoperative week	Group 1	95	95.50	0.00–0.06	1.000
	Group 2	95	95.50	0.00–0.06	
Neurological complications at 1-week postoperative follow-up	Group 1	95	95.50	0.00–0.00	1.000
	Group 2	95	95.50	0.00–0.00	

Group 1 = 9–14 years; Group 2 = 15–18 years. Data are presented as mean rank values obtained from the Mann–Whitney U test. *: *p* < 0.05 Abbreviation: VAS = Visual Analog Scale, nan–nan = Confidence interval not calculated as this is a multi-category variable.

**Table 3:** Comparison of the evaluated parameters between BMI groups.

Parameter	Group	n	Mean Rank	95% CI	*p*
Preoperative sedation requirement	Group A	76	111.50	0.69–0.87	0.000 *
	Group B	114	84.83	0.42–0.60	
Volume of local anesthetic used	Group A	76	77.53	2.50–2.70	0.000 *
	Group B	114	107.48	2.77–2.93	
Intraoperative sedation requirement	Group A	76	103.25	0.38–0.60	0.062
	Group B	114	90.33	0.27–0.44	
Motor block duration	Group A	76	97.64	130.15–136.43	0.658
	Group B	114	94.07	131.95–135.16	
Sensory block Duration	Group A	76	96.64	164.77–173.26	0.814
	Group B	114	94.74	166.92–172.38	
Intraoperative hypotension	Group A	76	98.25	0.05–0.18	0.182
	Group B	114	93.67	0.02–0.10	
Intraoperative bradycardia	Group A	76	97.25	0.01–0.11	0.150
	Group B	114	94.33	0.00–0.05	
Intraoperative nausea and vomiting	Group A	76	101.00	0.07–0.23	0.013 *
	Group B	114	91.83	0.01–0.09	
Postoperative nausea and vomiting	Group A	76	95.75	0.01–0.11	0.875
	Group B	114	95.33	0.01–0.09	
Postoperative hypotension	Group A	76	96.25	0.01–0.11	0.612
	Group B	114	95.00	0.01–0.07	
Post-dural puncture headache	Group A	76	95.00	0.01–0.09	0.735
	Group B	114	95.83	0.01–0.09	
Postoperative urinary retention	Group A	76	95.50	0.00–0.00	1.000
	Group B	114	95.50	0.00–0.00	
Adverse events observed after the first postoperative week	Group A	76	95.75	0.00–0.07	0.772
	Group B	114	95.33	0.00–0.05	
Neurological complications at 1-week postoperative follow-up	Group A	76	95.50	0.00–0.00	1.000
	Group B	114	95.50	0.00–0.00	

Group A = BMI 16.00–19.65 kg/m^2^; Group B = BMI 19.66–23.31 kg/m^2^. Data are presented as mean rank values based on the Mann–Whitney U test. *: *p* < 0.05.

## Data Availability

The data presented in this study are available on reasonable request from the corresponding author. The data are not publicly available due to privacy and ethical restrictions.

## Abbreviations

ASA	American Society of Anesthesiologists
VAS	Visual Analog Scale
BMI	Body mass index
CSF	Cerebrospinal fluid
PONV	Postoperative nausea and vomiting
mYPAS	Modified Yale Preoperative Anxiety Scale
SPSS	Statistical Package for the Social Sciences
